# Hepatitis B Vaccination in Advanced Chronic Kidney Disease: A Quality Improvement Project at a Veteran Affairs Chronic Kidney Disease Clinic

**DOI:** 10.3390/idr13040094

**Published:** 2021-12-06

**Authors:** Jacob Hettenbaugh, Ryan Mullane, Gayle Gillispie, Valerie Shostrom, Linda Flores, Jennifer A. Fillaus, Marius C. Florescu, Denise Murcek, Ketki K. Tendulkar

**Affiliations:** 1 Department of Internal Medicine, Division of Nephrology, University of Nebraska Medical Center, Omaha, NE 68138, USA; jacob.hettenbaugh@unmc.edu (J.H.); ryan.mullane@unmc.edu (R.M.); Gayle.Gillispie@va.gov (G.G.); vshostrom@unmc.edu (V.S.); Linda.Flores2@va.gov (L.F.); Jfillaus@omahanephrology.com (J.A.F.); mflorescu@unmc.edu (M.C.F.); denise.murcek@va.gov (D.M.); 2 VA Nebraska-Western Iowa Health Care System, Omaha, NE 68105, USA

**Keywords:** chronic kidney disease, hepatitis B vaccine, seroconversion, pre-dialysis

## Abstract

Hepatitis B vaccination is recommended in all patients with end-stage kidney disease (ESKD). However, only 50–60% of these patients achieve protective antibody levels if immunized after starting dialysis. Strategies to overcome this low seroconversion rate include a 6-month vaccination schedule starting earlier [chronic kidney disease (CKD) stage 4 and 5] to ensure immunity when patients progress to ESKD. We conducted a quality improvement program to immunize pre-dialysis patients. Patients who were found to have a negative baseline serology with a negative hepatitis B surface antibody level (HBsAb) were offered vaccination on a 6-month schedule (0, 1 and 6 months) with one of two available vaccines within the VA system (Recombivax™ or Engerix™). HBsAb titers were checked 3–4 months later, and titers ≥ 12 mIU/mL were indicative of immunity at VA. Patients who did not seroconvert were offered a repeat schedule of three more doses. We screened 198 patients (187 males and 11 females) with CKD 4 and 5 [glomerular filtration rate (GFR) < 29 mL/min/1.73 m^2^]. The median age of this cohort was 72 years (range 38–92 years). During the study period of 5 years (2015–2020), 10 patients were excluded since their GFR had improved to more than 30 mL/min/1.73 m^2^, 24 others had baseline immunity and 2 refused vaccination. The hepatitis B vaccination series was not started on 106 patients. Of the remaining 56, 12 patients progressed to ESKD and started dialysis before completion of the vaccination schedule, 6 expired and 1 did not come to clinic in 2020 due to the pandemic. Of the 37 patients who completed the vaccination schedule, 16 achieved seroconversion with adequate HBsAb titers, 10 did not develop immunity despite a second hepatitis B vaccination series, while 11 did not get a second series. Given the low seroconversion rate, albeit in a small cohort, vaccination should be considered in patients with earlier stages of CKD. Other options include studies on FDA approved vaccines of shorter duration. We plan to increase awareness among nephrologists, patients and nursing staff about the importance of achieving immunity against hepatitis B.

## 1. Introduction

The hepatitis B virus (HBV) is a known infectious risk in the chronic kidney disease (CKD) and end-stage kidney disease (ESKD) population. This patient population is at a higher risk of acquiring this virus, defined as positive hepatitis B surface antigen (HBsAg), compared to the general population [1] due to skin breaches, exposure to blood products, and sharing of dialysis machines. Despite the implementation of strict hygiene protocols to minimize risk, there is still a significant increased risk [2] of hepatitis B infection and potential subsequent increased risk of developing hepatocellular carcinoma (HCC) [3]. Chronic HBV infection in kidney transplant patients also lead to an increased risk of liver injury and cirrhosis [4].

In addition to the implementation of cleaning procedures, vaccination needs to be strongly considered. New cases of chronic HBV in the ESKD population have been reduced by 70% since vaccination efforts have been implemented, but the ability to mount adequate protective immune titers following vaccination is diminished compared to other healthy adults [5,6].

With the current challenges of vaccinating this population, there have been some studies on the optimal timing and dosing for HBV vaccination; however, limited data are available. Earlier investigators have found that those immunized at earlier stages of CKD were more likely to have a desired seroconversion [7]. Despite these promising results, there is no standard for the vaccination of CKD patients. There are also minimal data on the practical challenges associated with the initiation of hepatitis B vaccination prior to the onset of dialysis. In our study, we investigated the effectiveness of early efforts to vaccinate patients with CKD prior to initiating dialysis.

## 2. Patient and Methods

### 2.1. Patient Cohort

Patients with CKD stage 4 and 5 (GFR less than 29 mL/min/1.73 m^2^) were identified at the Chronic Kidney Disease (CKD) clinic at a single VA institution between 2015 and 2020. Their medical records were reviewed, including their hepatitis B serologic status. Data abstracted from the patient records included age, gender and GFR at study entry and during the study. Immune status was checked by measuring hepatitis B surface antibody (HBsAb) levels. This project was determined to be a quality improvement project by the institutional IRB and, hence, a waiver of informed consent was granted.

### 2.2. Vaccination

Patients who were HBsAb negative were offered education regarding the benefits of the hepatitis B vaccine and subsequent vaccination with one of two US FDA approved hepatitis vaccines (Engerix™ or Recombivax™ 40 mcg intramuscular injection) on a 6-month schedule (0, 1 and 6 months). HBsAb titers were checked 2–3 months after completion of the immunization schedule. The seroconversion was measured using a Hepatitis B Surface Antibody assay manufactured by Ortho Clinical Diagnostic. The data are presented using the cutoff levels cleared for use by the FDA in clinical laboratories. Negative is any value less than 5.00 mIU/mL and the patient was not considered to be immune to hepatitis B. Indeterminate is greater than or equal to 5.00 mIU/mL up to 11.99 mIU/ML, and it was recommended that testing be repeated in 2–3 weeks. Positive is any value greater than or equal to 12.00 mIU/mL and the patient was considered to be immunized to hepatitis B. Patients with anti-HBs ab ≥ 12 mIU/mL, were considered immune. Patients who did not achieve immunity were offered a booster dose and a second series if antibody titers were still inadequate.

### 2.3. Statistical Methods

An SAS version 9.4 was used for all summaries and analyses. For age and GFR, descriptive statistics, including minimum/maximum, quartiles, and median, are reported. The effect of age and GFR on the efficacy of seroconversion were compared using a nonparametric Mann–Whitney test.

## 3. Results

**Figure 1:**
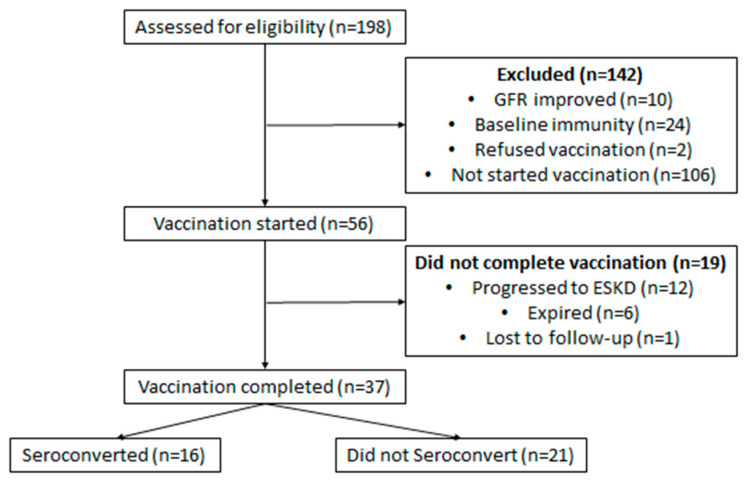
CONSORT Diagram.

A total of 198 CKD patients were identified during the study period (Figure 1). Of these, 10 were eliminated from further analysis, since their GFR was higher than 30 mL/min/1.73 m^2^. Twenty-four patients had a positive hepatitis B surface antibody at baseline, while two patients refused vaccination. Out of the remaining 162 eligible patients, 56 patients started their immunization schedule, while the vaccination series was not started on 106. These details are shown in Table 1.

Of the 56 patients who started vaccination, 12 patients progressed to ESKD and started dialysis before completion of the vaccination schedule, while 6 patients expired and 1 did not come to clinic in 2020 due to the COVID-19 pandemic. A total of 37 patients completed the vaccination schedule. 15 patients received a combination of Engerix and Recombivax, 12 received Recombivax, while 10 received Engerix. Of these, 16 (43%) achieved seroconversion with adequate HBsAb titers. The number of seroconverters was five (33%) with the combination, five (41.6%) with Recombivax and six with Engerix (60%). These differences were not statistically significant (*p* = 0.4). Ten patients (27%) did not seroconvert despite a second series of Hepatitis B vaccination, while eleven patients have not completed their second series thus far.

Neither age nor GFR at onset of vaccination were associated with seroconversion. The median age of patients who seroconverted was 69 years, compared to 71 years for those who did not (*p* = 0.12). Similarly, there was no difference in the GFR at onset of vaccination between seroconverters (24 mL/min/1.73 m^2^) and those who did not convert (18.5 mL/min/1.73 m^2^) (*p* = 0.12) (Table 1).

## 4. Discussion

This quality improvement study found multiple challenges with vaccinating patients with CKD against hepatitis B. The majority of patients were not able to start their vaccination series due to various system related issues. These included limited physician time in the clinic and lack of dedicated resources towards discussion regarding the risk/benefit ratio of hepatitis B vaccination. Of the patients who were able to complete their vaccination series, only 43% showed evidence of immunity against hepatitis B, suggesting a need for starting the process earlier in their CKD course.

HBV is an easily transmissible blood-borne pathogen that, despite having an overall low prevalence in the United States, can still cause seroconversion within dialysis centers [2]. Hemodialysis patients are at increased risk of transmission indirectly via possible contaminated surfaces, needles or medication [8,9]. Due to their compromised immunoreactivity, almost 60% of hepatitis B infected dialysis patients are at risk of becoming chronic carriers, which further emphasizes the increased viral transmission risk in dialysis units. While there have been advances in the treatment of hepatitis B with effective antiviral therapies, the treatment duration is long, possibly even lifelong [10]. Hence, there has been an emphasis on its prevention with the use of vaccinations. A critical component of any successful vaccination program is patient education regarding the long-term benefits [11]. Vaccination is a very cost-effective preventive measure and should be fully utilized.

Patients with ESKD requiring dialysis do not seem to mount an immune response following hepatitis B vaccination. There have been some studies that have shown a differential efficacy of the available vaccines in patients with end stage renal disease. Studies have shown that Engerix may be more effective in this population [12]. We did not find a significant difference between the three cohorts, although Engerix had a numerically higher number of seroconverters. This may be due to the small sample size of our population. Factors that impair the proper immune response in this population include uremia from inadequate dialysis, poor nutrition, anemia and co-morbid conditions such as diabetes mellitus. A meta-analysis of CKD studies that included four studies with 332 dialysis patients showed that while their seroconversion rates did not differ, antibody titers were higher in the seroconverters vaccinated with a double dose of the hepatitis B vaccine [13].

One possible approach to increasing seroconversion is to develop new vaccines that target different moieties on antigen presenting cells. Toll-like receptors (TLR) are a class of pattern recognition receptors found on antigen-presenting cells such as dendritic cells or macrophages. The stimulation of these receptors with antigens may lead to the development of an adequate adaptive response, which is the goal premise of most vaccinations. Several new HBV vaccines have shown promising results in early clinical trials, as they contain adjuvants that help to stimulate these specific TLRs and help achieve seroprotection [14]. Small studies have shown promising seroconversion rates with two novel vaccines. One of these is HB-AS04, a novel vaccine that contains HBV surface antigen paired with a monophosphoryl lipid. A molecule absorbed on aluminum salt (AS04) showed promise in a small clinical trial of HD-dependent non-responders to an initial HBV vaccination attempt who seroconverted after a single dose [15]. HB-AS02, an investigational vaccine that contains the HBsAg combined with a detoxified derivative of the lipopolysaccharide molecule found on the cell wall of Salmonella minnesota and QS21, an immunostimulant extracted from the Chilean soapbark tree, showed a seroconversion rate of 77% of CKD patients that did not develop an adequate response to conventional hepatitis B vaccine series [15]. A third novel vaccine, HBsAg-1018, using TLR9 combined with an oligonucleotide as an adjuvant, showed that an increased proportion of patients seroconverted, not only in the healthy population but also with the CKD population [15].

Additional measures that can be implemented and may improve efficacy include intradermal injections, as this may more effectively stimulate dendritic cells and promote a more effective adaptive immune response [16]. Investigations into adjuvants that are not contained within the vaccine itself have also been considered. These approaches include topical imiquimod administration before receiving an intradermal HBV vaccine [17] or co-administration of oral levamisole [18], which has demonstrated increased seroconversion with HBV vaccination due to the promotion of B lymphocyte function and stimulation of depressed T cell activity.

In advanced CKD patients, there is a lack of response by the immune system, which continues to be a cause of both low rates of seroconversions and high infectious complications [19]. Furthermore, it remains important to achieve adequate seroconversion prior to kidney transplantation, whether pre-emptive or not, as the immunosuppression regimen further makes this difficult.

Impaired monocyte functioning results in inadequate antigen presentation to the antigen-presenting cells, generating weakened memory cells and inadequate antibody production after vaccination. These disturbances are mostly noted in CKD stages 4 and 5. Additional research would be beneficial for improving the rates of seroresponsiveness to vaccines in this vulnerable population. Adherence to the available vaccination schedules is highly required in the CKD population to decrease morbidity and mortality in vaccine preventable illnesses [20].

Ahmadi found that there was no significant difference in achieving seroprotection between four doses of a 40 µg compared to three doses of a 20 µg of the Euvax B [21]. Alternative formulations have also been investigated, including one reviewed by Fabrizi, who looked at the effectiveness of Fendrix, a recombinant HBV vaccine with an improved adjuvant system, and found promising results [22].

The leading explanation for this is an impaired cellular response due to chronic inflammation. A proposed surrogate is CD40, which has been shown to inhibit immunoglobulin production in vitro. Patients on chronic hemodialysis are noted to have an average of five-fold higher concentrations of CD40 compared to the general population, whereas CKD patients have a three-fold higher level compared to the general population [11]. Chronic uremia has also been noted to impair cellular and humoral immunity via the impaired production of T cell costimulatory molecules [23]. Investigators have also noted an impaired seroconversion rate with HBV vaccination in patients with diabetes mellitus compared to non-diabetic patients of comparable stages of CKD [24]. Additional variables have been identified in a recent meta-analysis including low albumin, low hemoglobin and low PTH [25]. Low levels of Vitamin D deficiency have also been implied as a significant and independent factor for difficulty with seroconversion, which can happen in advanced CKD. Unfortunately, in this study, the correction of vitamin D levels had no influence on immunologic reactivity [26].

Logistic issues, including a lack of physician time in clinic for discussion and counseling regarding the benefits of hepatitis B vaccination, the coordination of nursing injection visits with other clinic appointments and missed nursing injection appointments over a 6-month period, were the main challenges to completing the vaccinations in this project. A major limitation of this project is the small number of patients who completed the vaccination series. This may have been responsible for the lack of significance in the differences in the seroconversion rate seen among patients who received the different hepatitis B vaccines (*p* = 0.12). Informing nephrologists of the importance of the timing of hepatitis B vaccination, especially the need to vaccinate CKD patients prior to the onset of dialysis, may lead to an increased uptake of this practice. These issues need to be considered in further research in this area.

## 5. Conclusions

Given the low seroconversion rate, albeit in a small cohort, studies should be conducted using other vaccine formulations available, even in the CKD patient population. Another option would be to vaccinate patients with earlier stages of CKD to increase the rate of immunity should they progress to ESRD. It is critical to increase awareness among nephrologists and nursing staff about the importance of this. Our study also emphasizes the need for active patient involvement and improved health literacy.

## Figures and Tables

**Table 1:** Factors affecting seroconversion.

Variable	Converters N = 16	Non-Converters N = 21	*p*-Value
Age–Median (25th, 75th)	69 (58, 75)	71 (69, 77)	0.1203
GFR–Median (25th, 75th)	18.5 (15, 23)	24 (16, 25)	0.2070

## Data Availability

The data that support the findings of this study are restricted based on US federal guidelines governing patient care, and so are not publicly available. De-identified data are, however, available from the authors upon reasonable request and with the permission of the US Government.

## Figure Legends

Figure 1: CONSORT Diagram showing the patient flow in the project.
